# Development of a Microemulsion Formulation for Antimicrobial SecA Inhibitors

**DOI:** 10.1371/journal.pone.0150433

**Published:** 2016-03-10

**Authors:** Jiahuai Hu, Nagaraju Akula, Nian Wang

**Affiliations:** Citrus Research and Education Center, Institute of Food and Agricultural Sciences, University of Florida, Lake Alfred, Florida, United States of America; Fujian Agriculture and Forestry University, CHINA

## Abstract

In our previous study, we have identified five antimicrobial small molecules via structure based design, which inhibit SecA of *Candidatus* Liberibacter asiaticus (Las). SecA is a critical protein translocase ATPase subunit and is involved in pre-protein translocation across and integration into the cellular membrane in bacteria. In this study, eleven compounds were identified using similarity search method based on the five lead SecA inhibitors identified previously. The identified SecA inhibitors have poor aqueous solubility. Thus a microemulsion master mix (MMX) was developed to address the solubility issue and for application of the antimicrobials. MMX consists of N-methyl-2-pyrrolidone and dimethyl sulfoxide as solvent and co-solvent, as well as polyoxyethylated castor oil, polyalkylene glycol, and polyoxyethylene tridecyl ether phosphate as surfactants. MMX has significantly improved the solubility of SecA inhibitors and has no or little phytotoxic effects at concentrations less than 5.0% (v/v). The minimum inhibitory concentration (MIC) and minimum bactericidal concentration (MBC) of the SecA inhibitors and streptomycin against eight bacteria including *Agrobacterium tumefaciens*, *Liberibacter crescens*, *Rhizobium etli*, *Bradyrhizobium japonicum*, *Mesorhizobium loti*, and *Sinorhizobium meliloti* phylogenetically related to Las were determined using the broth microdilution method. MIC and MBC results showed that the 16 SecA inhibitors have antibacterial activities comparable to that of streptomycin. Overall, we have identified 11 potent SecA inhibitors using similarity search method. We have developed a microemulsion formulation for SecA inhibitors which improved the antimicrobial activities of SecA inhibitors.

## Introduction

Citrus greening or Huanglongbing (HLB) is a destructive disease worldwide and drastically reduces citrus production in Florida [1]. In the U.S., HLB is associated with Gram-negative bacterium *Candidatus* Liberibacter asiaticus (Las) [2, 3]. Las has not been cultured *in vitro* and is transmitted through grafting or Asian citrus psyllid (*Diaphorina citri*). HLB-diseased trees have led to premature fruit drop and poor quality fruit with bitter flavor. The typical symptoms of Las-infected citrus trees include yellow shoot, yellow leaf with blotchy-mottling and severe vein corking as well as small-size and lopsided fruit[4, 5]. Las-infected branches die back and the infected trees decline and die within several years of symptom expression [6]. Meanwhile, HLB disease is difficult to manage due to the lack of resistant cultivars. Removal of HLB diseased trees, replanting, psyllid control with insecticides combined with foliar nutritional spray [7] led to substantial increase in production costs and threatened the economic sustainability of citrus production in Florida. Several new tactics such as thermotherapy [8] and antibiotic treatment [9] are under development to protect new planting and rescue HLB-diseased trees. Antibiotics such as streptomycin and tetracycline have not been approved for HLB management in citrus, though they are available for other fruit crops such as apple and peach [10]. Currently, there are growing interests in identifying effective antibacterial agents for citrus HLB management.

Protein secretion in bacteria is a critical and complex process and also the main virulence mechanism of many pathogenic bacteria [11, 12]. SecA is a conserved and essential protein in all bacteria, hence it is considered as a promising target for development of antibacterials [13]. SecA is a peripheral membrane ATPase, which involves in pre-protein translocation across and integration into the cellular membrane in bacteria [13–15]. In our previous studies we have identified five small molecule inhibitors against SecA based on virtual screening of small molecule compounds from ZINC database [16, 17]. We have been aiming to identify more potent antimicrobial SecA inhibitors using different approaches, e.g., similarity search method. Similarity search method plays an important role in lead-discovery programs in the pharmaceutical and agrochemical industries [18]. Similarity search involves taking a molecule with the required activity, then searching the target structure against a database to find the molecules that are most similar to it [18, 19].

One limitation of the identified SecA inhibitors is poor aqueous solubility. It is critical to develop suitable formulations for the SecA inhibitors to increase their antimicrobial efficacy. Formulating water insoluble compounds was a key component in the process of developing effective antimicrobial treatment. Microemulsion is defined as ‘a system of water, oil and amphiphile which is a single optically isotropic and thermodynamically stable liquid solution’ [20]. The appearance of microemulsion is transparent and its droplet size ranges from 10 to 100 nM [21]. Microemulsion system significantly reduces the use of organic solvents, but generally requires higher concentrations of surfactants to reduce interfacial tensions [20, 22]. Microemulsions of pesticides were more effective, stable, and environmental friendly than other formulations such as emulsion concentrate, suspension concentrate, and wettable powders [23–26].

Briefly, in this study, we reported our recent progress in identifying new antimicrobial SecA inhibitors using similarity search method. We have developed a microemulsion formulation for SecA inhibitors and tested antibacterial activity of the identified compounds. The identified antimicrobial SecA inhibitors provide candidates for further optimization and alternatives for treatment of HLB-diseased trees.

## Materials and Methods

### Ethics statement

No specific permission was required to run this study in greenhouse and field trials. Our field studies did not involve endangered or protected species.

### Computational methodology

Similarity search method was used for virtual screening against ZINC database. In our search criteria, the five SecA inhibitor structures identified previously [16] were used as reference, with 90% identity as constraint. Homology model of SecA [18] was used for molecular docking study. The ligand and protein preparations were done using ligprep and protein preparation wizard of Maestro package (Maestro, version 9.9, Schrödinger, LLC, New York, NY, 2014). The grid generation necessary for docking was done within Glide program [27]. A receptor grid was created around the ATP present in the binding site. Receptor grid files were generated by excluding ATP with outer grid set to 20 Å^3^ with an inner box (10 × 12 × 10) Å^3^ and extended 1Å at a time in xyz orientations. The rotation of hydroxyl groups of the binding site residues was allowed for all the grids without any constraints. Glide XP flexible docking was carried out on the ligands with 10 poses per ligand being stored and 2 poses per molecular structure were used for analysis. All the molecular modeling studies have been performed on HP ProLiant, RedHat Linux operating system and the docking postures were taken using PyMOL program [28].

### Chemical compounds

The selected sixteen compounds were procured from ChemBridge (San Diego, CA) & Interbioscreen (Chernogolovka, Russia). Solvents of methanol, ethanol, dimethyl sulfoxide, N-dimethyl-2-pyrrolidone, N-octyl-2-pyrrolidone, propylene carbonate, cyclohexanone, propylene glycol mono-methyl ether acetate, 1, 2-cyclohexane dicarboxylic acid di-isononyl ester, isoparaffin, and hexane were purchased from Sigma-Aldrich Co. (St. Louis, MO) or Thermo Fisher Scientific (Waltham, MA). Hallcomid M-8-10 was procured from Stepan (Northfield, IL). Akzo Nobel Surface Chemistry (Chicago, IL) provided emulsifier Emulpon^™^ CO-360, CO-550, Witconol PEG-400, NP-200, Witconate P-1220EH, and Amadol 5195. The Dow Chemical Company provided Tergitol L-61, L-62, 15-S-30, and 15-S-9. Rhodia provided Rhodafac RE 610 and RS 410. Croda Inc (Edison, NJ) provided samples of ATLAS G-5000-SO-(AP). All chemical compounds were stored at room temperature under lab conditions.

### Solvent and surfactant screening

The solubility of HPLC-grade SecA inhibitors at 25°C and 65°C was evaluated in a test tube containing 2 g of solvent. The solvents that completely dissolved the inhibitors and form “oil solution” were chosen for MMX development. The MMX was composed by mixing equal weights of “oil solution” and surfactants in a water bath at 65°C for 4 h. After cooling down to room temperature, the suitability of a surfactant was determined by the appearance of the sample. Thereafter, suitable surfactants were mixed in different ratios to determine the best combination. The optimum combination of surfactants was determined by the sample appearance, turbidity, phase separation, the temperature range over which transparency was maintained, and low temperature stability.

### Microemulsion preparation

To further stabilize microemulsion, Hallcomid M-8-10 (dimethyl-octanamide & dimethyl-decanamide) and ETR (the optimum combination of surfactants Emulpon CO-360, Tergitol L-61, and Rhodafac RE-610) were mixed at weight ratios of 1/7, 2/7, 3/7, 5/7, and 7/7. The resulting solution was blended with SecA inhibitor “oil solution” at weight ratio of 4:1, 3:2, 1:1, 2:3, and 1:4. The dilution characteristics was assessed by diluting each sample with distilled water, tap water, and a hard water containing Ca^2+^ and Mg^2+^ at the concentration of 180 mg/ml. The dilution series consisted of six concentrations of 0.5, 1.0, 2.5, 5.0, 7.5, and 10% (v/v). Other physicochemical characteristics such as the appearance, and centrifugation properties were also obtained. The stability of microemulsion was checked by centrifuging at 8000 rpm for 10 min at room temperature.

### Phytotoxicity assessment of MMX microemulsion

Four experiments were conducted to evaluate whether solvents and surfactants in MMX microemulsion had any phytotoxic effect on seed germination and root elongation, 1-year-old Hamlin seedlings, and 5-year-old Hamlin trees. A total of eight concentrations of MMX tested were 0.0, 0.5, 1.0, 2.5, 5.0, 7.5, 10%, and 20% (v/v). In the germination experiment, seeds extracted manually from in-season ripe Hamlin fruit (*Citrus* × *sinensis*) were surface-sterilized in a 1.0% sodium hypochlorite solution for 10s, rinsed with distilled water, then placed on Whatman No. 1 filter paper in a plastic box. The filter paper was moistened with 15 ml of distilled water or MMX solutions at six concentrations as described above. Each concentration was replicated 4 times and each replicate had 40 seeds evenly spaced on the moist paper. The germination box was incubated in a dark growth chamber at 28°C. The filter paper was replenished with 4 mL of distilled water or MMX solution at each concentration on a 3-day interval. A seed was scored as germinated when the radicle protruded over 2 mm. Three-weeks after germination, the stem and primary root of the seedlings were measured for their length and fresh weight.

In the greenhouse evaluation, 1-year-old Hamlin seedlings were sprayed at 4-day interval for 2 weeks with five MMX concentrations of 0.0, 0.5, 1.0, 2.5, and 5.0% (v/v). Each treatment consisted of 3 seedlings. All treated seedlings were visually inspected daily for any toxicity response from the first spray till 2-week after the last spray. In the field trial, 5-year-old Hamlin trees were sprayed or injected with MMX solutions of SecA inhibitors at five concentrations aforementioned. Each concentration was replicated with 3 trees. Compressed air sprayer (handheld, 2L) was used for foliar spray. Trunk injection was carried out using tree I.V. Micro Infusion^®^ (Arborjet Inc., MA). Briefly, two holes per tree were made on the main stem 30 cm directly above root flares to facilitate best uptake and canopy distribution. Holes were drilled to a depth of 2–3 cm using 7.14 mm (9/32”) drill bit; a No. 3 Arborplug^®^ was set into each hole for proper seal with Arborplug^®^ setter and a rubber hammer. All treated trees were visually inspected for any toxicity response 2-week after the treatment.

### Bacterial strains and their culturing conditions

Cultures of *Agrobacterium tumefaciens* and *Escherichia coli* strain DH5α were maintained in LB medium at 28°C and 37°C, respectively; *Liberibacter crescens* [29] and *Xanthomonas citri* subsp. *citri* [30] were grown in BM7 medium and nutrient broth respectively, at 28°C. *Rhizobium etli* (ATCC 51251) [31], *Bradyrhizobium japonicum* (ATCC 10324) [32], *Mesorhizobium loti* (ATCC 700743) [33], and *Sinorhizobium meliloti* (RM1021) [34] were grown in Yeast-Mannitol broth at 28°C. Bacterial culture was grown to the logarithmic-phase to match the turbidity of 0.5 McFarland standard [35]. Thereafter, the bacterial suspensions were adjusted to approximately 10^6^ CFU/mL with appropriate medium.

### MIC and MBC determination

MIC and MBC were estimated using the broth microdilution method [35, 36]. A 2-fold dilution series ranging from 1024 to 8 μg/mL were prepared in medium broth for SecA inhibitors and streptomycin (positive control), in which MMX concentrations decreased in 2-fold from 1.024 to 0.008% (V/V). Therefore, a similar 2-fold dilution series of MMX without any compound was included to determine whether MMX interacted with SecA inhibitors. Aliquots of each dilution (100 μL per well) was transferred to 4 replicated wells in a 96-well microtiter plate. Thereafter, a total of 100 μl of inoculum suspension was added to each well and mixed with a micropipette. The incubation temperature for all bacteria were 28°C, except for *E*. *coli* at 37°C. The incubation periods for *E*. *coli*, *A*. *tumefaciens*, *X*. *citri* subsp. *citri*, *S*. *meliloti*, *M*. *loti*, *R*. *etli*, *B*. *japonicum*, and *L*. *crescens* were 1, 1, 2, 2, 2, 4, 4, and 5 days, respectively. After incubation, each well in a MIC plate was examined for visible bacterial growth as evidenced by turbidity. The MIC is defined as the lowest concentration that inhibits visible growth of a microorganism [35, 37]. Turbidity of MIC plates was also recorded using a microplate spectrophotometer at a wavelength of 630 nm (Bio-Rad Benchmark Plus, Hercules, CA). MBC was determined by re-inoculating inhibitor-free agar plates with 100 μl of culture samples from the first cloudy well and all clear wells. The inoculated plates were incubated under aforementioned growth conditions. MBC is defined as the lowest concentration with the reduction by 99.9% of the initial inoculum of multiplying bacteria. The experiments were repeated 3 times on different dates.

## Results

### Identification of SecA inhibitors using similarity search method

**Fig 1:**
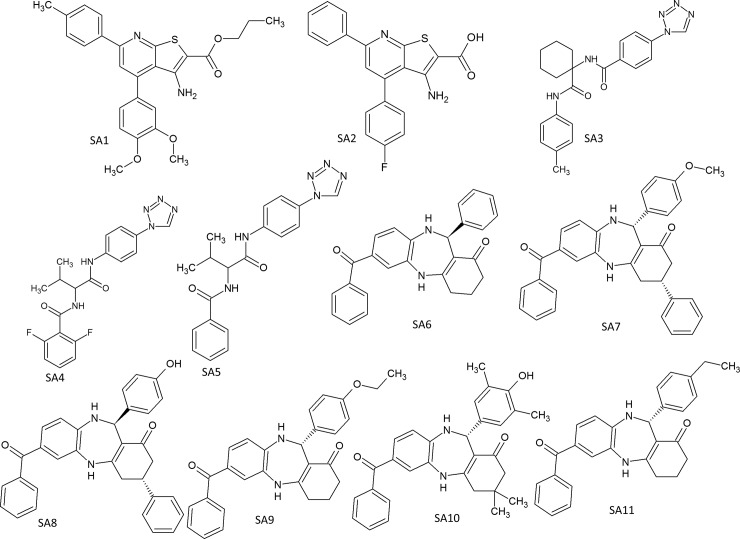
Structural information of the 11 antimicrobial SecA inhibitors identified using similarity search method.

A total of 565 structures were identified in our initial screening with 90% identity of five lead structures reported previously (Fig 1 C16-C20). Different grid size files were generated at ATP binding site for molecular docking study. Grid box dimensions were 14 × 12 × 14 Å^3^ in xyz directions from ATP binding site. The docked ATP molecule retained its crystal structure orientation and the extended grid size was relatively bigger than our previous grid file used in structure based design. Higher grid size resulted in exploring more pockets at the binding site. Glide XP docking method was used to select the best docked structures based on molecular docking scores. Among 565 structures that were initially identified, we have selected 163 structures, whose docking scores are greater than -6.5k.cal/mole. In addition, these 163 structures were visually inspected with their docking conformations. We have chosen 40 compounds for antibacterial study. However, only 11 compounds were commercially available to purchase from different chemical suppliers. The structural information of the 11 identified compounds was given in Fig 1 (SA1-SA11). These 11 compounds are structurally similar to C17, C18, or C20.

### SecA inhibitors, solvents, surfactants, and MMX

The names, structures, and membrane-related chemical properties of the sixteen potential antimicrobial SecA inhibitors were summarized in Table 1. The molecular weight of these compounds ranged from 337 to 526 and the molecular properties of all sixteen compounds were compatible with Lipinski’s rule of five [38], indicating their good membrane permeability and other biological activities. However, these small molecules have poor aqueous solubility. The solubility of these compounds were generally low in all but two solvents. Moderate solubility of 5–10 g/L was obtained with N-dimethyl-2-pyrrolidone and dimethyl sulfoxide. When N-dimethyl-2-pyrrolidone was mixed with dimethyl sulfoxide in a ratio of 1:1 (v/v), a greater solubility of these inhibitors attained 20 g/L. The concentration of these compounds was maintained at 20 g/L for all the subsequent assays. Surfactants Emulpon CO-360, Tergitol L-61, and Rhodafac RE-610 were able to form a single continuous phase with transparent appearance (Table 2). The best ratio of Emulpon CO-360, Tergitol L-61, and Rhodafac RE-610 (ETR) were 6:1:1 because this ratio produced the sample with best low temperature stability (Table 3). Hallcomid M-8-10 is an excellent emulsifier and solvent that can improve stability of formulations such as emulsion concentrate and microemulsion and may also inhibit crystallization of active ingredient from organic phase upon dilution with water. When Hallcomid M-8-10/ETR/ “oil solution” ratio was 1:7:2 (w/w/w), the optimal composition of MMX was obtained because it exhibited transparent appearance and did not form any precipitate. Moreover, no phase separation was noted after centrifugation at 8000 g for 10 min at room temperature. When the MMX was diluted with distilled water, tap water, and hard water to the concentrations of 0.5, 1.0, 2.5, 5.0, 7.5, and 10% (v/v), a clear microemulsion was formed without any phase separation observed. All samples remained uniform and clear solution without floating oil and any precipitation after standing at the room temperature for 4 h. After storage at 4°C for 4 weeks, no phase separation or precipitate formation was observed, indicating good physiochemical stability.

**Table 1:** Names, formulas, and chemical properties of sixteen compounds tested in this study.

Compounds	MW^u^	logP^v^	HBD^w^	HBA^x^	NRB^y^	TPSA^z^
C16	337	3.5	2	4	2	83.6
C17	436	5.0	2	6	3	103.9
C18	420	3.5	2	6	6	111.0
C19	384	4.7	2	4	4	79.9
C20	400	3.9	2	2	3	58.2
SA1	526	4.6	1	10	8	111.0
SA2	364.4	5.01	2	4	3	80
SA3	510	5.0	1	9	7	102.0
SA4	400.4	1.93	2	8	6	101
SA5	364.4	1.89	2	8	6	101
SA6	394.5	5.12	2	4	3	58
SA7	500.6	6.74	2	5	5	67
SA8	486.6	6.21	3	5	4	78
SA9	438.5	5.56	2	5	5	67
SA10	476.6	6.31	3	5	3	78
SA11	466.6	6.04	2	4	4	58

^u^ MW = molecular weight

^v^ logP = oil/water distribution coefficient

^w^ HBD = hydrogen bond donors

^x^ HBA = hydrogen bond acceptor

^y^ NRB = number of rotatable bonds

^z^ tPSA = topological polar surface area.

**Table 2:** Results of surfactant screening assay.

Surfactants	Appearance at 10-min standing at 25°C	Appearance after 4-h standing at 25°C
Emulpon CO-360	Transparent	Transparent
Emulpon CO-550	White emulsion	Phase separation
ATLAS G-5000-S0-(AP)	Semitransparent	Phase separation, precipitation
Tergitol L-61	Transparent	Transparent
Tergitol L-62	Semitransparent	Phase separation
Tergitol 15-S-9	Turbid	Phase separation, precipitation
Tergitol 15-S-30	Turbid	Phase separation, precipitation
Witconol PEG-400	White emulsion	Phase separation, precipitation
Witconol NP-200	White emulsion	Phase separation, precipitation
Witconate P-1220EH	Turbid	Phase separation, precipitation
Amadol 5195	White	Phase separation, precipitation
Rhodafac RS-410	Semitransparent	Phase separation
Rhodafac RE-610	Transparent	Transparent

**Table 3:** Optimization results of three component surfactant system containing different weight ratios of Emulpon CO-360, Tergitol L-61, and Rhodafac RE-610.

ETR ratio (w/w/w)	Appearance		Stability at 4°C	Cloud point (°C)^z^
	0 h	4 h		
6:1:1	Transparent	Transparent	Transparent	>70
6:2:1	Transparent	Phase separation	Transparent	>70
6:3:1	Transparent	Transparent	Phase separation	>70
2:6:1	Turbid	Not tested	Not tested	Not tested
3:6:1	Turbid	Not tested	Not tested	Not tested
4:6:1	Semitransparent	Phase separation	Not tested	Not tested

^z^ cloud point is the temperature at which the sample became turbid, which was determined by increasing the temperature of a water bath.

### Phytotoxicity assessment of MMX

Seed germination and seedling growth were affected by MMX in a concentration-dependent manner. Seeds treated with 5% or lower MMX started to germinate 1-week after incubation in the dark at 28°C. Maximum germination rate of 84.4% was observed in seeds receiving 0.5% MMX treatment, but was not significantly different from that of non-treated seeds or seeds treated with 1.0% and 2.5% MMX (Table 4). MMX at the concentration of 5.0% significantly reduced germination rate and completely inhibited seed germination at 10% or higher concentrations. Similarly, the growth of primary roots and stems of germinated seeds was not affected by 1% MMX or below (Table 4). In the presence of MMX at the rate of 2.5%, the seedling growth was significantly reduced and concentrations of 5.0% or above produced a more accentuated inhibitory effect on seedling growth (Table 4). In particular, stem growth was inhibited to a greater extent when compared to root elongation.

**Table 4:** Effects of various concentrations of MMX on germination, root length, stem length, as well as root and stem fresh weight of *Citrus* × *sinensis^z^*.

Concentrations	Germination (%)	Length (mm)		Fresh weight (mg) of primary root and stem
		Primary root	Stem	
0.0	76.3 a	28.5 a	22.0 a	40.8 a
0.5	84.4 a	27.3 a	18.5 a	35.2 a
1.0	71.3 a	27.2 a	17.6 a	33.4 a
2.5	62.5 a	23.9 b	12.6 b	29.1 b
5.0	26.9 b	16.8 c	6.9 c	12.1 c
7.5	13.8 b	12.3 c	5.2 c	6.9 d
10.0	1.9 c	4.0 d	0.0 d	0.0 e
20.0	0.0 c	0.0 d	0.0 d	0.0 e

^z^ all seeds were surface-sterilized in a 1.0% sodium hypochlorite solution and germinated at 8 concentrations of MMX (0.0%-20.0%, V/V) in a dark growth chamber at 28°C. Three weeks into germination, the number of germinated seeds was recorded, root and stem were excised and their length and fresh weigh were measured. All values were means of 4 replicates and each replicate consisted of 40 seeds. All numbers were reported as mean. One-way ANOVA and Duncan’s multiple range test was used to determine whether there were significant differences (*P* < 0.01) among 8 concentrations of MMX. Numbers followed by the same letters indicated no significant difference.

When 1-year-old Hamlin seedlings in the greenhouse or 5-year-old Hamlin trees in a citrus grove were sprayed or injected with MMX at the concentration range from 1.0% to 5.0%, toxic response or physical injuries such as burning and stunting were not observed on the leaves and stems of any MMX-treated plants. However, discoloration was observed on the portion of treated scions (up to water line) soaked overnight in 5.0% MMX, indicating possible toxic effect of MMX on scions at higher concentrations.

### MICs and MBCs of antimicrobial compounds

The antibacterial activities of sixteen compounds were assessed using eight bacterial species, including *L*. *crescens* and *Rhizobium* spp. closely related to Las (Table 5). *L*. *crescens* and *B*. *japonicum* represent slow-growing bacteria with doubling time of 36 h [29] and 20 h [32], respectively. *E*. *coli* and *A*. *tumefaciens* were fast-growing bacteria with doubling time of 31–46 min [39]and 105 min, respectively. The other four bacteria had doubling time between these two groups. In general, all SecA inhibitors had greater inhibitory effects on slow-growing bacteria than fast-growing bacteria. For example, the MICs and MBCs for *A*. *tumefaciens* and *E*. *coli* were two-fold to several-fold greater than *L*. *crescens*, *R*. *etli*, and *B*. *japonicum* (Table 5). While there were some variations in MICs and MBCs among sixteen SecA inhibitors against 8 bacteria, the overall differences were relatively small and all compounds tested exhibited good inhibitory activities against all but *E*. *coli* (Table 5). Specifically, MICs and MBCs for *L*. *crescens* ranged from 16 to 128 μg/mL, from 32 to 128 μg/mL, respectively; MICs and MBCs for *X*. *citri* subsp. *citri* ranged from 16 to 64 μg/mL and from 32 to 64 μg/mL, respectively; MICs and MBCs for four *Rhizobium* bacteria ranged from 8 to 32 μg/mL and from 6 to 64 μg/mL, respectively; MICs and MBCs for *E*. *coli* ranged from 256 to 512 μg/mL, and >512 μg/mL, respectively; MICs and MBCs for *A*. *tumefaciens* ranged from 32 to 512 μg/mL, and from 128 to >512 μg/mL, respectively; MICs and MBCs of streptomycin against all bacterial species were in the ranges from 32 to 512 μg/mL, from 64 to >512 μg/mL, respectively.

**Table 5:** MICs and MBCs of sixteen antimicrobial SecA inhibitors against bacteria (μg/mL).

Compounds^x^	*L*. *crescens*		*R*. *etli*		*B*. *japonicum*		*M*. *loti*		*S*. *meliloti*		*X*. *citri* subsp. *citri*		*A*. *tumefaciens*		*E*. *coli*	
	MIC	MBC	MIC^y^	MBC^z^	MIC	MBC	MIC	MBC	MIC	MBC	MIC	MBC	MIC	MBC	MIC	MBC
C16	32	32	32	64	32	64	16	32	16	32	16	32	64	64	256	512
C17	16	16	32	32	16	16	16	32	32	64	16	32	32	128	512	512
C18	16	16	32	64	64	64	16	32	32	64	32	64	32	128	512	512
C19	32	32	32	64	16	64	8	16	16	16	16	32	32	128	256	512
C20	16	32	32	32	32	32	8	16	32	64	16	32	64	128	512	512
SA1	32	32	32	64	16	32	16	32	16	32	32	64	32	64	512	512
SA2	32	32	32	64	64	128	16	32	16	32	32	64	32	64	512	512
SA3	32	32	32	64	32	32	16	32	32	64	32	64	32	128	512	512
SA4	32	32	32	64	32	32	16	32	16	32	64	128	32	64	512	512
SA5	16	32	64	64	32	64	16	32	32	64	64	128	32	64	512	512
SA6	32	64	16	32	16	16	16	32	32	64	32	64	16	64	512	512
SA7	16	32	32	32	32	32	16	32	32	64	64	128	32	64	256	512
SA8	32	64	64	64	32	32	16	32	32	32	64	128	16	64	256	512
SA9	32	32	32	32	32	32	16	32	16	32	32	64	16	64	512	512
SA10	32	32	16	32	32	32	16	32	16	32	32	64	32	128	256	512
SA11	32	64	16	32	32	64	16	32	16	32	32	64	32	64	512	512
STM	32	32	16	16	32	32	16	32	64	128	32	32	64	128	128	256
MMX^w^	0.26	>0.5	0.26	>0.5	0.26	>0.5	>0.5	>0.5	>0.5	>0.5	>0.5	>0.5	>0.5	>0.5	>0.5	>0.5

^w^ A 2-fold dilution series of MMX without SecA inhibitors ranged from 1.024 to 0.008% (V/V).

^x^ A 2-fold dilution series ranging from 1024 to 8 μg/mL of SecA inhibitors were prepared in medium broth, in which the initial concentration of MMX was 1.024% (V/V).

^y^ MIC is the lowest concentration that inhibits visible growth of a microorganism after incubation.

^z^ MBC is defined as the lowest concentration with the reduction by 99.9% of the initial inoculum of multiplying bacteria.

## Discussion

**Fig 2:**
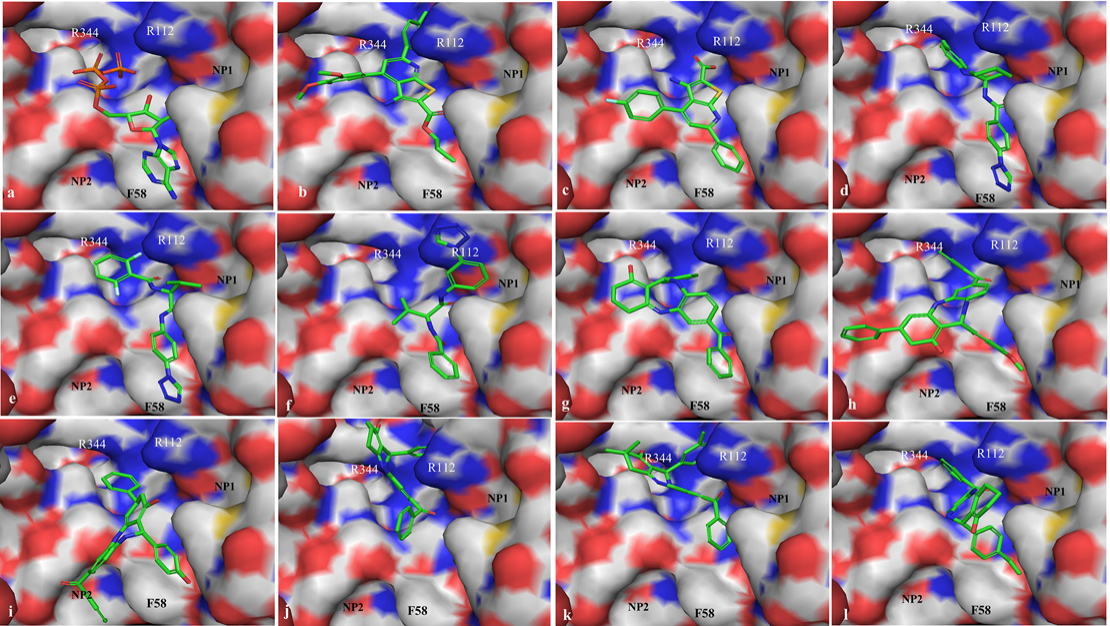
SecA of Las ATP Binding site is displayed as molecular surface interface and labeled the critical residues Phe58, Arg112, Arg344 on the surface of the model. NP1, NP2 are the newly identified pockets at the ATP binding site of SecA. The docked ligands were represented in stick model and they are a).ATP, b).SA1, c).SA2, d).SA3, e).SA4, f).SA5, g).SA6, h).SA7, i).SA8, j).SA9, k).SA10 & l).SA11. PyMOL program was used to build the protein surface models.

Similarity search method has been successfully used to identify antimicrobial small molecule lead compounds [18]. In the current study, a total of 11 SecA inhibitors have been identified from different chemical databases based on similarity search to the five SecA inhibitors identified in our previous study [40, 41]. These 11 newly identified compounds have shown antibacterial activity against different bacterial strains (Table 5). However, the 11 compounds showed comparable, but not improved antibacterial activities compared to 5 SecA inhibitors identified previously [40, 41] (Table 5). This may indicate the limitation of the similarity search method in optimization of lead compounds. The newly identified 11 compounds were classified into three categories: SA1-SA2 (Class I-Thieno-pyridine derivatives), SA3-SA5 (Class II-Tetrazole derivatives), and SA6-SA11 (Class III-Benzodiazepin derivatives) (Fig 1) based on their structures. Identification of binding sites and analysis of binding pockets play an import role in structure based drug design [42]. Notably, the docking results revealed two additional binding pockets besides ATP binding orientation, since the extended grid used was not limited to ATP binding sites. The newly identified pockets were defined as NP1 and NP2 (Fig 2A–2l). Binding site cavities and intermolecular interactions were critical for drug development [43]. Therefore, the docked ligands were analyzed to compare their conformations against ATP, orientation towards newly identified sites and inter molecular interactions with critical binding site residues (π-π interactions with aromatic ring of F58, Hydrogen donor and H-bond formation of R112 and R344 with H-N-H). When compared the docking poses of class I structures (SA1 & SA2) with ATP (Fig 2A), SA1 retained the ATP binding orientation (Fig 2B), but lost π-π interactions with F58. SA2 had π-π interactions with F58 and H-bond interactions with R112 and R344 except triphosphate site was not completely filled by SA2 structure (Fig 2C). Class II compounds (SA3 & SA4) had similar conformations as ATP and partially oriented towards the newly identified site NP1 (Fig 2D and 2E). SA5 had better binding at NP1 site but lost the binding mode at triphosphate site (Fig 2F). Among the six Class III compounds, only two (SA6 & SA8) of them had π-π interactions with F58 and partial inter molecular interactions with R112 & R344 (Fig 2G and 2I). One of the structural fragments of SA8 completely occupied NP2 site (Fig 2i). Compared to ATP, all the six structures (SA6-SA11) had similar orientation but weaker intermolecular interactions (Fig 2H and 2I). None of the 11 compounds had all inter molecular interactions with activity site residues or binding pockets occupancy, which may be partially responsible for the lack of enhanced activities. Nonetheless, the additional binding sites identified in this study provide more options for future screening of effective molecule against HLB. Our results suggest that designing a small molecule that resides in binding pockets NP1 & NP2 along with the ATP binding site could be an effective approach. Enhanced binding may be attained by adding groups inserted deeper into these hydrophobic pockets (NP1 & NP2).

Citrus HLB is a destructive disease worldwide. In the U.S., HLB is associated with Gram-negative bacteria *Candidatus* Liberibacter asiaticus (Las). Identification of effective antibacterial agents provides an alternative management approach to revive HLB diseased trees. Successful antibacterial treatment against Las requires effective antibacterial agents, systemic translocation and suitable residual effect. While several antibiotics were found effective in suppressing Las titers in graft-based screening assays, phytotoxicity and poor translocation of antibiotics were also noted for some of these antibiotics [9, 44]. This scenario supports the great needs to develop an efficient vehicle that can deliver the active antibacterials to the Las target in a tree canopy. We have developed a microemulsion formulation for SecA inhibitors and demonstrated antibacterial activity comparable with streptomycin. This microemulsion is physically stable and insensitive to changes in temperature and ionic strength. When used at rates less than 2.5% (v/v), it did not cause any phytotoxic effect on seed germination, seedling growth, and 1-year-old or older citrus plants. These results indicated that microemulsion formulation of our antimicrobial compounds could be used to suppress the disease in an existing Las-infected tree. It offers a valuable alternative option for managing citrus HLB disease for prolonged productivity. It is not clear whether MMX formulation of SecA inhibitors could be degraded easily in a citrus tree and residue analysis in fruit should be performed in future studies.

This study identified the suitable combinations of solvents and surfactants that can be used to develop microemulsion of SecA inhibitors. It has been demonstrated that three component surfactant system was more efficient and flexible than a single or couple of surfactants [45–47]. Both solvents and surfactants used in this study were environmentally friendly, readily biodegradable, and commonly used in pesticide formulations. For example, Hallcomid M-8-10 is an excellent solvent, cosolvent, and emulsifier that is commonly found in pesticide formulations. Emulpon CO-360, a castor oil ethylene oxide adduct with 36 units of ethoxylation, is a non-ionic emulsifier with hydrophilic-lipophilic balance ratio of 13.4; Tergitol L61 (polyether polyol), an ethylene oxide/propylene oxide copolymers, is a non-ionic emulsifier with hydrophilic-lipophilic balance ratio of two. Rhodafac RS 610, a polyoxyethylene tridecyl ether phosphate, is an anionic emulsifier and penetrant with pH buffering capability. It has been shown that the surface charge from anionic surfactants generated repulsive forces between droplets and therefore greatly improve the stability of emulsion [48]. All 4 surfactants enhanced wettability, dispersing ability, and penetration ability of the SecA inhibitor formulation, which is critical for an efficient delivery of SecA inhibitor to Las target in a tree canopy. Microemulsion formulation of a pesticide was reported to have better control efficiency than other pesticide formulations, including emulsion concentrate and suspension concentrate [24, 25, 49].

The microemulsion formulation of SecA inhibitors developed in this study exhibited good physiochemical stability over a wide range of ionic strength and temperature. This could be explained by the non-ionic surfactants used in the preparation of microemulsion because they are less sensitive to variations in pH and ionic strength. This result was confirmed by higher cloud point and excellent microemulsion formulation with distilled water, tap water and hard water. Cloud point is a key indicator of the physical stability. As temperature increases, the hydrophilic-lipophilic balance and hydrophilicity of a surfactant will decrease. When temperature reaches a critical point, it results in phase separation and transition of appearance from transparency to opaque. It has been shown that the concentrations of Mg^2+^ and Ca^2+^ had significant effect on hydrophilic-lipophilic balance values of surfactants in a microemulsion system [50]. An earlier report indicated that nonionic surfactant blends formed thin aqueous films between oil droplets and oil phase, thus preventing oil droplets from coalescing into homophase [51]. Hallcomid M-8-10 further stabilized microemulsion by reducing interfacial tension, adjusting the hydrophilic-lipophilic balance value and blending with other surfactants [52]. The viscosity, droplet size and uniformity, and effective concentration of SecA inhibitors in the formulation remains to be characterized in the future study.

Microemulsion formulation greatly enhanced the antibacterial activity of SecA inhibitors. For example, when these compounds were dissolved in DMSO and diluted with sterile distilled water, their MIC values against *A*. *tumefaciens* ranged from 128 to 256 μg/mL [16]. However, when the compounds were formulated as microemulsion, their antimicrobial effect was improved approximately 4-fold with MIC values ranging from 32 to 64 μg/mL against *A*. *tumefaciens* (Table 5). This result indicated that microemulsion of SecA inhibitors had a significantly stronger inhibitory effect than did an aqueous DMSO solution. The increased inhibitory effect was likely due to several factors: 1) solvent mixtures of NMP and DMSO could partially contribute to the enhanced antibacterial activity, as documented in previous reports [53]; 2) the improved permeability due to smaller droplet size in a microemulsion formulation; 3) the presence of multiple surfactants and penetrants results in higher penetrability, much larger contact area of the active substance to the target sites of SecA.

In conclusion, we have identified 11 SecA inhibitors using similarity search method. We have developed a microemulsion formulation for SecA inhibitors which improved the antimicrobial activities of SecA inhibitors.
